# Urinary peptidomic liquid biopsy for non-invasive differential diagnosis of chronic kidney disease

**DOI:** 10.1093/ndt/gfad200

**Published:** 2023-09-11

**Authors:** Emmanouil Mavrogeorgis, Tianlin He, Harald Mischak, Agnieszka Latosinska, Antonia Vlahou, Joost P Schanstra, Lorenzo Catanese, Kerstin Amann, Tobias B Huber, Joachim Beige, Harald D Rupprecht, Justyna Siwy

**Affiliations:** Mosaiques Diagnostics GmbH, Hannover, Germany; Institute for Molecular Cardiovascular Research (IMCAR), RWTH Aachen University Hospital, Aachen, Germany; Mosaiques Diagnostics GmbH, Hannover, Germany; Mosaiques Diagnostics GmbH, Hannover, Germany; Mosaiques Diagnostics GmbH, Hannover, Germany; Center of Systems Biology, Biomedical Research Foundation of the Academy of Athens, Athens, Greece; Institut National de la Santé et de la Recherche Médicale (INSERM), U1297, Institute of Cardiovascular and Metabolic Disease, Toulouse, France; Université Toulouse III Paul-Sabatier, Toulouse, France; Department of Nephrology, Angiology and Rheumatology, Klinikum Bayreuth GmbH, Bayreuth, Germany; Kuratorium for Dialysis and Transplantation (KfH) Bayreuth, Bayreuth, Germany; Friedrich-Alexander-University Erlangen-Nürnberg, Erlangen, Germany; Department of Nephropathology, Institute of Pathology, Friedrich-Alexander-University of Erlangen-Nürnberg, Erlangen, Germany; III. Department of Medicine, University Medical Center Hamburg-Eppendorf, Hamburg, Germany; Hamburg Center for Kidney Health (HCKH), University Medical Center Hamburg-Eppendorf, Hamburg, Germany; Department of Infectious Diseases/Tropical Medicine, Nephrology/KfH Renal Unit and Rheumatology, St Georg Hospital Leipzig, Leipzig, Germany; Kuratorium for Dialysis and Transplantation (KfH) Renal Unit, St Georg Hospital, Leipzig, Germany; Department of Internal Medicine II, Martin-Luther-University Halle/Wittenberg, Halle (Saale), Germany; Department of Nephrology, Angiology and Rheumatology, Klinikum Bayreuth GmbH, Bayreuth, Germany; Kuratorium for Dialysis and Transplantation (KfH) Bayreuth, Bayreuth, Germany; Friedrich-Alexander-University Erlangen-Nürnberg, Erlangen, Germany; Mosaiques Diagnostics GmbH, Hannover, Germany

**Keywords:** chronic kidney disease, differential diagnosis, peptides, UMAP, urine

## Abstract

**Background and hypothesis:**

Specific urinary peptides hold information on disease pathophysiology, which, in combination with artificial intelligence, could enable non-invasive assessment of chronic kidney disease (CKD) aetiology. Existing approaches are generally specific for the diagnosis of single aetiologies. We present the development of models able to simultaneously distinguish and spatially visualize multiple CKD aetiologies.

**Methods:**

The urinary peptide data of 1850 healthy control (HC) and CKD [diabetic kidney disease (DKD), immunoglobulin A nephropathy (IgAN) and vasculitis] participants were extracted from the Human Urinary Proteome Database. Uniform manifold approximation and projection (UMAP) coupled to a support vector machine algorithm was used to generate multi-peptide models to perform binary (DKD, HC) and multiclass (DKD, HC, IgAN, vasculitis) classifications. This pipeline was compared with the current state-of-the-art single-aetiology CKD urinary peptide models.

**Results:**

In an independent test set, the developed models achieved 90.35% and 70.13% overall predictive accuracies, respectively, for the binary and the multiclass classifications. Omitting the UMAP step led to improved predictive accuracies (96.14% and 85.06%, respectively). As expected, the HC class was distinguished with the highest accuracy. The different classes displayed a tendency to form distinct clusters in the 3D space based on their disease state.

**Conclusion:**

Urinary peptide data present an effective basis for CKD aetiology differentiation using machine learning models. Although adding the UMAP step to the models did not improve prediction accuracy, it may provide a unique visualization advantage. Additional studies are warranted to further validate the pipeline's clinical potential as well as to expand it to other CKD aetiologies and also other diseases.

## Graphical Abstract

**Graphical Abstract:**
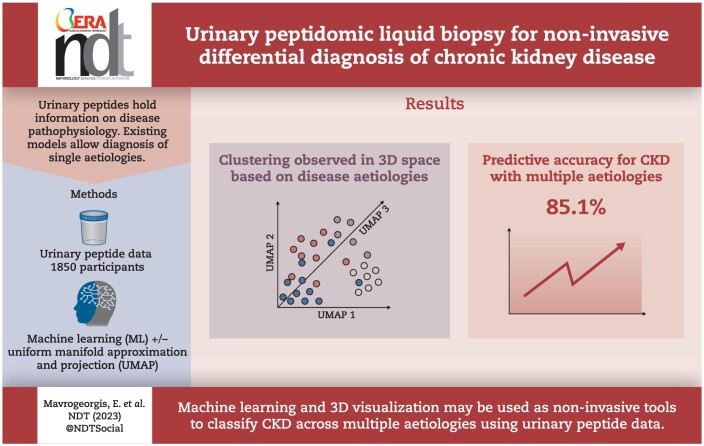

KEY LEARNING POINTS **What was known:**

- Kidney biopsy is considered the gold standard for determining the aetiology of chronic kidney disease (CKD).
- The only diagnostic markers that allow sparing a kidney biopsy are combined serum domain antibodies (e.g., phospholipase A2 receptor) for membranous nephropathy.
- Genetic test applicability is limited to cases of genetic variants with varying performance.

**This study adds:**

- Differentiation of multiple aetiologies is possible with good accuracy by applying novel machine learning algorithms to thousands of exactly defined urinary peptides.
- Individual sample spatial visualization can be performed, forming distinct clusters that reflect disease state through the application of the uniform manifold approximation and projection algorithm on urinary peptide data.

**Potential impact:**

- The presented non-invasive differentiation and visualization approach could be used in clinical practice to support diagnostic decisions.
- The approach could be applied not only for the CKD aetiologies presented, but also potentially to additional CKD aetiologies.
- With the proper design, it could also allow for a non-invasive robust e.g., disease monitoring or treatment response prediction, thus supporting therapeutic decisions.

## INTRODUCTION

The high prevalence and economic burden [1] of chronic kidney disease (CKD) underscore the need for further efforts to address its associated challenges. CKD exhibits many aetiologies and considerable heterogeneity, making it a complex, multifaceted condition and a diagnostic challenge. Failure to

identify CKD in early stages, where therapy is expected to lead to optimal outcome, eventually results in an advanced disease state, in which irreversible kidney damage has already occurred. A major clinical concern relates to the differential diagnosis of different CKD aetiologies, mostly relying on an invasive kidney biopsy as the gold standard, despite its limitations. Since biopsy is an invasive procedure with potential complications like associated potential bleeding [2], non-representative sampling, disagreement in the interpretation between pathologists [3] and dependence on appropriate organ size, the implementation of specific non-invasive biomarkers that could support diagnosis and selection of therapy appears highly relevant. In addition, repeated biopsies (to assess treatment response or disease progression) are generally not possible.

Several efforts have been made to identify biomarkers that could non-invasively support the CKD differential diagnosis. We investigated the literature using the terms: ‘chronic kidney disease’, ‘CKD’, ‘kidney disease’, ‘differential diagnosis’, ‘types’, ‘aetiolog*’, ‘etiolog*’, ‘classifier*’ and ‘panel’. This literature search indicated that research on CKD differential diagnosis appears to be mainly focused on genetic studies and CKD-related genetic panels [4–12]. These studies at times confirmed the presence of suspected inherited kidney diseases [9] and even led to a correction of the traditional diagnosis [7]. However, adult CKD might not always be attributed to hereditary origin. In contrast, urinary protein–based markers, being closer to the phenotype, could be of clinical relevance.

Glazyrin *et al*. [13] demonstrated that using urinary proteomics, patients with nephrosclerosis could be distinguished from patients with mixed diabetic kidney disease (DKD) and glomerulonephritis, with the latter two being subsequently differentiated from each other using plasma samples. Although displaying high classification performance, the study was based on only 34 participants, and the performance was not evaluated in an independent dataset. Validation was partly performed by Fernando *et al*. [14], focusing only on differentiating CKD of unknown aetiology from a mixed CKD aetiology class (DKD, nephrosclerosis, glomerular diseases).

The analysis of urinary peptides based on capillary electrophoresis coupled to mass spectrometry (CE-MS) has been extensively applied for the identification and assessment of biomarkers in a number of diseases [15–18]. The robustness of CE-MS has been highlighted in several studies [19–22]. Using the thousands of peptides identified in urine for developing machine learning models based on support vector machine (SVM) algorithms has demonstrated superior performance in comparison with the state of the art [23]. Additionally, several disease-specific SVM-based peptide models have been established in the field of CKD, such as the IgAN237 [24] or CKD273 [25], the latter being recognized with a letter of support from the US Food and Drug Administration [26] and implemented in a clinical trial for early detection of DKD [27].

In a first attempt to non-invasively identify different CKD aetiologies, Siwy *et al*. [28], using a cohort of 1180 participants, developed distinct models for seven CKD aetiologies, representing the current state of the art. The individual model performances reached an area under the curve of 0.77 or higher in the receiver operating characteristic curves using an independent test set. Although an aetiology-specific model could demonstrate substantial prediction of the targeted aetiology, potentially conflicting positive results produced by multiple single-aetiology models could result in an ambiguous diagnosis. Thus, a common classifier for distinguishing multiple aetiologies of a disease simultaneously appears highly clinically relevant. Novel algorithms, such as the uniform manifold approximation and Projection (UMAP) [29, 30], have since shown a promising variety of applications in biological data interpretation based on the ability to utilize a dataset's omic information (e.g., RNAs) for embeddings in a low-dimensional space.

Building on the available 1850 urine peptidomic datasets obtained from the Human Urinary Proteome Database [17], our aim was to establish a pipeline for the non-invasive differential diagnosis of CKD aetiologies in a novel approach, harnessing the dimensionality reduction and visualization capabilities of UMAP in a proof-of-concept study.

## MATERIALS AND METHODS

### Subjects and datasets

**Figure 1:**
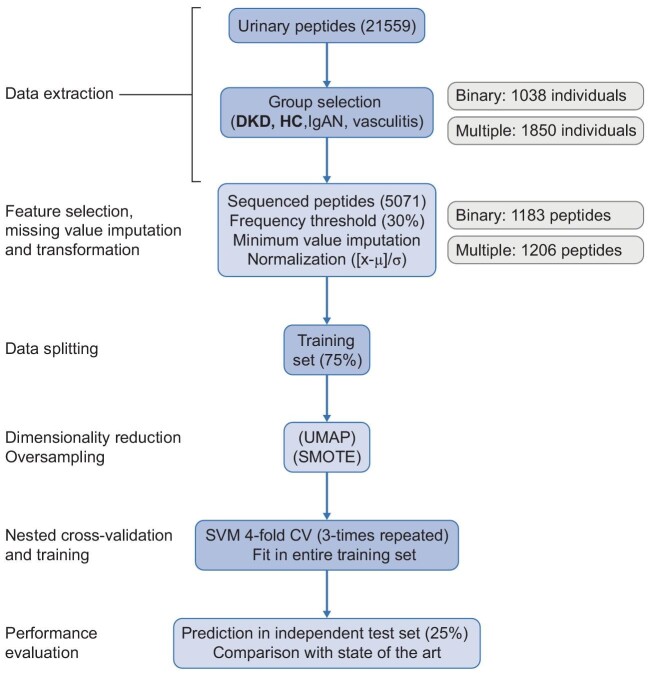
Study design. The urinary peptide datasets of a cohort of 1850 HC and CKD (DKD, IgAN and vasculitis) individuals were implemented into a supervised machine learning pipeline for classification based on disease (or lack thereof). The pipeline was performed separately for DKD and HC classes (binary classification) as well as all classes (multiclass classification). Initially, a splitting of the classification data into a training (75%) and a test (25%) set was performed. Then, the sequenced peptides present in at least 30% of the respective participants, were considered for further analysis and normalized in the training and test sets {[x-mean(x)]/standard deviation(x), considering the training set} after missing peptide values of each dataset were imputed based on the respective minimum values. A dimensionality reduction with the UMAP algorithm was performed (or skipped), while as an additional step during the training procedures in the multiclass classification only, the oversampling algorithm SMOTE [31] was applied. The latter produced synthetic participants in all classes until a certain ratio of the (initially) majority class (i.e., IgAN) was achieved, so as to account for the class imbalance. During a three-times repeated four-fold CV, SVM models were trained (in three out of four folds of the training set) and their performance was recorded (on the remaining fold) along the lines of an iterative search that relied on a Bayesian optimization [35] of the hyperparameters. The model that achieved the highest average accuracy across all the CV folds was selected as having the optimal combination of hyperparameter values. Subsequently, the selected model was trained in the entire training set and then tested for its predictive accuracy in the independent test set. μ, feature mean; σ, feature standard deviation; SMOTE, Synthetic Minority Over-sampling Technique; CV, cross-validation in training set.

Anonymized peptidomic data of 1850 urine samples corresponding to healthy controls (HC) and CKD patients of various aetiologies were extracted from the Human Urinary Proteome Database [17]. The HC samples were derived from participants without signs of CKD or significant loss of kidney function (estimated glomerular filtration rate ≥60 mL/min/1.73 m^2^) (*n* = 504). The CKD samples were derived from participants diagnosed with one of the following CKD aetiologies: immunoglobulin A nephropathy (IgAN) (*n* = 737), DKD (*n* = 534) and vasculitis (*n* = 75). The study design is depicted in Fig. 1.

All datasets were from previously published studies and fully anonymized. Diagnosis of IgAN and vasculitis were based on kidney biopsy. Diagnosis of DKD was generally assigned based on the clinical parameters. Only for six patients were results from biopsy available, in each case supporting the DKD diagnosis. The studies respected the regulations for protecting participants in medical research and the Declaration of Helsinki (2013). This study was approved by the ethics committee of the Friedrich-Alexander Universität Erlangen-Nürnberg, Germany (ethic approval code 264_20 B for the nephrological biobank and ethic approval code 221_20 B for the urinary proteomics analysis).

### Urine samples and CE-MS analysis

The methods used in this study are described in detail in the Appendix. All datasets used were from samples collected in the morning, after voiding the first urine. Samples were frozen within 6 h and stored at below –20°C. Stability and reproducibility of this process was extensively investigated and described in previous studies [19, 20, 22], demonstrating that urine samples stored >10 years at –20°C did not show any significant change in peptide content. Urine samples were analysed using CE-MS, peptide sequencing and data evaluation was performed as described (Appendix p. 1). In brief, peptides and proteins <20 kDa were separated in the CE based on their electrophoretic mobility and then ionized through electrospray. Subsequently, these ions were separated by a mass analyser based on their mass to charge ratio, before their relative abundance was detected. Only sequenced peptides present in at least 30% of the participants were used as an input for classification, being processed and normalized as described in Fig. 1.

### Machine learning

A machine learning pipeline was implemented to develop models that enable determining a diagnosis (class) of a participant using solely urinary peptidomics. For this non-invasive approach, models towards the following classifications (Appendix pp. 3–4) were developed: DKD and HC (binary) as well as DKD, HC, IgAN and vasculitis (multiclass).

To adjust for imbalance (due to different sample sizes) between the multiple diagnosis classes, random synthetic participants were introduced in each class until all were equally numbered reaching the ratio of the majority class (i.e., IgAN), as described [31]. The binary and multiclass classifications were performed both with and without applying the UMAP algorithm [29, 30] (Appendix pp. 2–3 [32, 33]) to the urinary peptides before they were used as an input to the SVM models. The UMAP algorithm performs dimensionality reduction, i.e. transforms the features (peptides) into a low-dimensional space (e.g., three dimensions). This is expected to potentially remove irrelevant (‘noise’) information of the data, while reducing complexity and the required analysis time. UMAP was applied onto the input data and the generated 3D space coordinates were used to plot the samples as single data points. The naïve (default hyperparameters), unsupervised (without considering the diagnosis information of the samples) UMAP as well as the naïve, supervised (considering the diagnosis information of the samples) and lastly, the tuned (selecting the specific UMAP hyperparameter values that led to the best SVM model classification results), supervised UMAP applications were considered for visually exploring the respective impact in terms of distinct diagnosis cluster formation in the UMAP plots.

To objectively assess the model performance, the dataset was randomly split into training and test sets based on sample groups in a 75:25 ratio for classification purposes (Appendix pp. 3–4). The training set was used to train candidate models (differing on their hyperparameter values that determined, e.g., the model's tolerance for misclassifications), with the goal of optimal diagnosis based on the peptide relative abundance. This training was performed for each candidate model based on the cross-validation (CV) method: the (training) set was randomly divided into four parts and each model was trained using the participants of the three parts and its performance was assessed in the fourth one. The model with the best average performance across all four different combinations was considered for further analyses. Lastly, after fitting in the entire training set, the accuracy of that model was estimated by assessing its performance in the independent test set. Since the test set is irrelevant to the training procedures it represents an unbiased source for assessing the model performance. This procedure was performed for each classification (w/o UMAP for binary/multiclass classifications). The multiclass classification models were compared with the ones developed by Siwy *et al*. [28]. The machine learning pipeline was based on R statistical software (Appendix pp. 4–5).

## RESULTS

Urinary peptidomic data of 1850 samples were extracted from the Human Urinary Proteome Database [17]. This set included 504 HC participants, 534 patients with DKD, 737 with IgAN and 75 with vasculitis, for whom the available clinical information is presented in Table 1. The study design is illustrated in Fig. 1. Applying a frequency threshold of 30% and limiting the analysis to sequenced peptides only, the subsequent steps were based on 1183 or 1206 peptides (for binary and multiclass classifications, respectively).

**Table 1:** Cohort clinical characteristics.

	DKD (*n* = 534)	HC (*n* = 504)	IgAN (*n* = 737)	Vasculitis (*n* = 75)
Age (years)	63.11 (12.37)	44.4 (18.33)	42.79 (14.69)	59.44 (14.36)
eGFR (mL/min/1.73 m^2^)	47.93 (25.36)	94.11 (17.51)	60.26 (30.79)	47.62 (30.85)
BMI (kg/m^2^)	29.88 (5.60)	27.19 (5.49)	26.35 (3.99)	25.17 (2.97)
dBP (mmHg)	76.96 (10.77)	78.93 (10.17)	85.86 (12.48)	80.92 (12.49)
sBP (mmHg)	142.96 (20.17)	134.54 (20.62)	135.95 (18.82)	139.88 (22.62)
uACR	888.23 (2487.13)	8.55 (6.38)	1241.3 (1431.66)	806.12 (885.92)
Male (%)	58.47	52.78	66.57	47.54

Given is the number of the participants of the entire classes. For the clinical characteristics each time a mean (standard deviation) or percentage is displayed, as calculated based on the available participant clinical information.

BMI, body mass index; dBP, diastolic blood pressure; eGFR, estimated glomerular filtration rate; uACR, urinary albumin to creatinine ratio; sBP, systolic blood pressure.

### Binary classification: differentiation of DKD and HC classes

**Figure 2:**
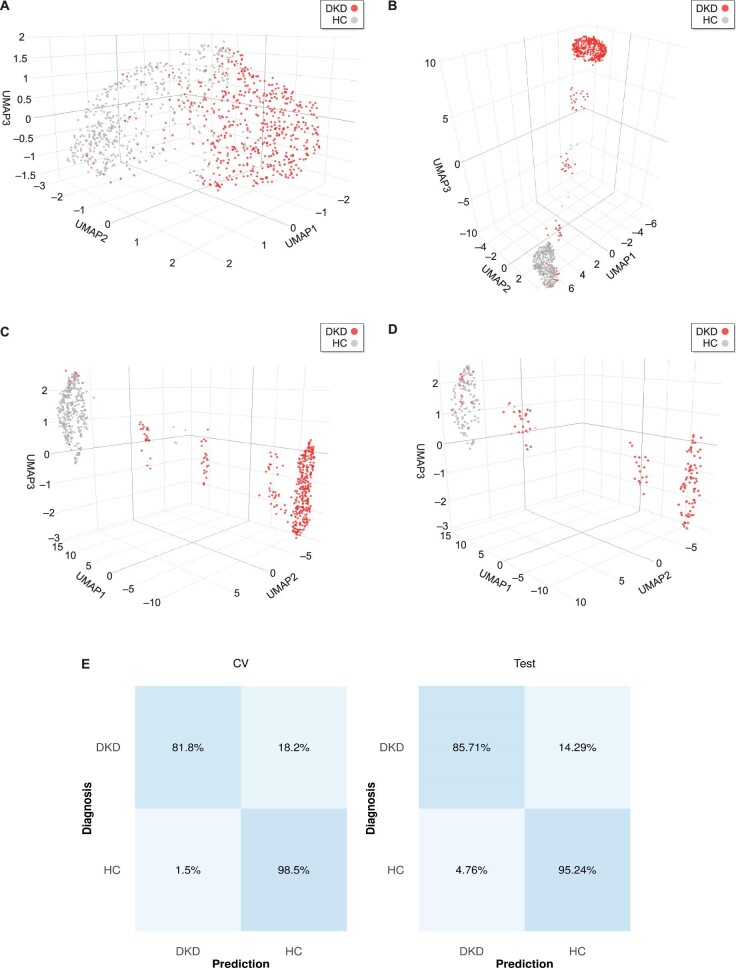
Binary classification results. The peptidomic profiles of DKD (red) and HC (gray) participants were used as a basis for the default hyperparameters of the UMAP algorithm in its (**A**) unsupervised as well as (**B**) supervised version. Cluster formation was more evident when the supervised UMAP with tuned parameters was performed, as observed in the (**C**) training set and (**D**) independent test set embeddings. (**E**) Confusion matrices based on the results of the training set cross-validation (CV, average across all folds) as well as the predictions in the independent test set. Classification accuracies are displayed in percentages.

Initially, UMAP was applied as a naive unsupervised dimensionality reduction method to the peptidomic data of 534 DKD and 504 HC participants to visualize their potential separation in the 3D space (Fig. 2A). Although the majority of the patients of the same class diagnosis appeared to be clustered, a substantial overlap of the clusters prevented clear separation. That observation indicated the utility of UMAP in embedding high-dimensional urinary peptidomic data in a low-dimensional space, but also that a supervised UMAP approach may be better suited for class separation. Therefore, supervised UMAP was applied, leading to a major class separation improvement in both its naïve (Fig. 2B) and tuned version (Fig. 2C and D). The selected UMAP-SVM model achieved 89.89% average accuracy in the cross-validated training set, while an overall 90.35% accuracy in the independent test set. The UMAP embeddings of the training and test sets are illustrated in Fig. 2C and D, respectively. Per-class accuracies of the model for both the cross-validated training set and the independent test set are illustrated in Fig. 2E.

### Multiclass classification: differentiation of multiple CKD aetiologies and HC classes

**Figure 3:**
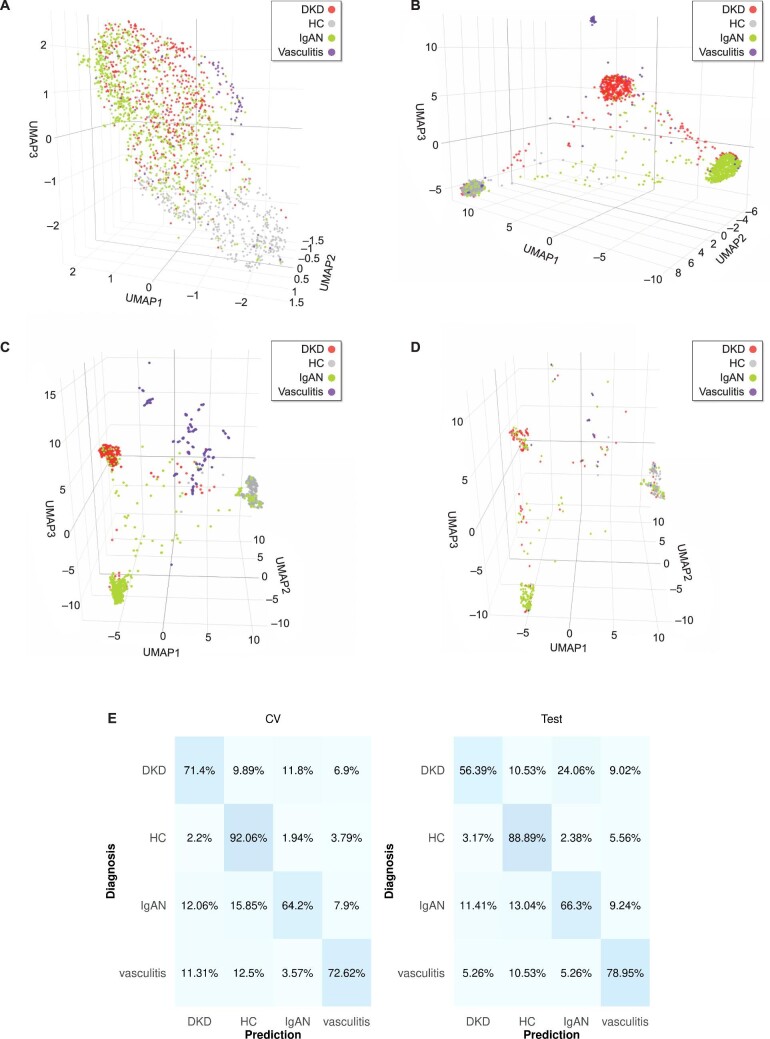
Multiclass classification results. The peptidomic profiles of DKD (red), HC (gray), IgAN (green) and vasculitis (purple) participants were used as a basis for the UMAP algorithm (default hyperparameters) in its (**A**) unsupervised as well as (**B**) supervised version. Cluster formation was more evident when the supervised UMAP with tuned parameters was performed, as observed in the (**C**) training set and (**D**) test set embeddings. (**E**) Confusion matrices based on the results of the training set cross-validation (CV, average across all folds) as well as the predictions in the independent test set. Classification accuracies are displayed in percentages. Of note, an oversampling step was performed during the training procedures.

Subsequently, the same pipeline was utilized to differentiate all four classes: DKD, HC, IgAN and vasculitis. Again, applying the naïve UMAP algorithm, a related tendency, but not clear cluster formation was observed (Fig. 3A). This was substantially improved in the respective supervised (naïve and tuned) UMAP embeddings (Fig. 3B–D). To adjust for the numeric imbalance of these classes, an oversampling approach [31] was implemented during the training procedures. The overall performance of the selected model in the cross-validated training set (average of 74.18%) as well as the predictions in the independent test set (70.13%) were recorded. In detail, predictions in the independent test set displayed accuracies of 56.39%, 66.30% and 78.95% for DKD, IgAN and vasculitis classes, respectively, achieving the highest accuracy (88.89%) in differentiating the HC class from CKD aetiologies (Fig. 3E).

### Comparison with SVM-only model

**Figure 4:**
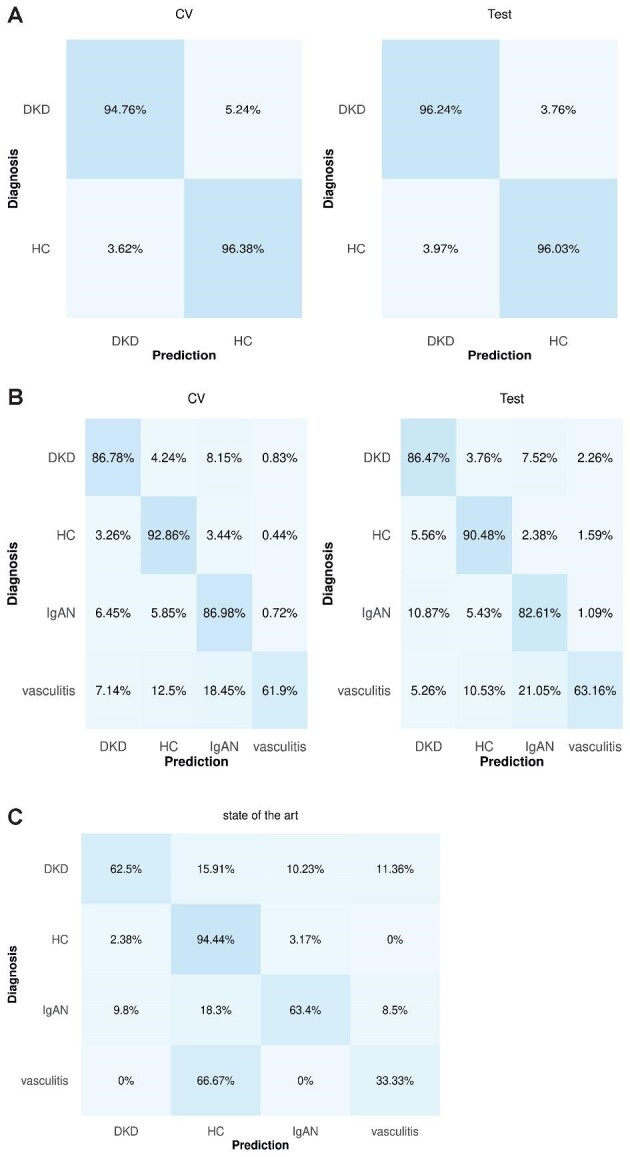
Comparison without including UMAP in the pipeline as well as with the current state of the art. Confusion matrices of the predictions in (**A**) binary and (**B**) multiclass classifications. (**C**) Predictions using the current state-of-the-art single-aetiology models [25, 28].

To evaluate the added value of UMAP as an important dimensionality reduction step in urinary peptidomics as well as the proposed pipeline as a whole, additional comparisons were performed. Initially, a SVM model was built and trained as described above, but skipping the UMAP step. In the binary classification, the selected model displayed an overall accuracy of ≥95.56% in both the cross-validated training set and in the independent test set (Fig. 4A). In the multiclass classification, the model achieved an overall average accuracy of 87.51% in the cross-validated training set, while the overall accuracy in the independent test was 85.06%, with the per-class accuracies of 86.47%, 82.61% and 63.16% for DKD, IgAN and vasculitis, respectively (Fig. 4B). Of note, in the latter classification, the HC class was distinguished with 90.48% accuracy.

### Comparison with the state of the art in CKD urinary proteomics

Subsequently, the comparison with the individual CKD-aetiology models described in Siwy *et al*. [28] was performed. The models specific for DKD (and nephrosclerosis), IgAN and vasculitis classes were considered since these aetiologies were relevant in the current study. Predictions were made only for the 373 participants of the independent test set (*n* = 462) that had not been a part of the training set of the CKD differential diagnosis models developed by Siwy *et al*. [28]. These corresponded to: 88 DKD, 126 HC, 153 IgAN and 6 vasculitis individuals. To differentiate HC from CKD patients, the CKD273 [25] model was utilized. The models correctly predicted 62.50%, 94.44%, 63.40% and 33.33% of the DKD, HC, IgAN and vasculitis classes, respectively (Fig. 4C).

## DISCUSSION

In the current work, we demonstrated that the assessment of CKD-specific aetiologies is possible with good accuracy, using an artificial intelligence–driven approach by applying the SVM algorithm on urinary peptides. The presented findings demonstrate that this non-invasive approach could be used as an alternative/complementary way within the context of CKD diagnosis. We also explored whether adding a novel dimensionality reduction/visualization algorithm (UMAP) to ‘transform’ the information (relative abundance) of these peptides into three only spatial coordinates could actually improve the classification performance (diagnosis) as well as illustrate the samples as single data points in the 3D space.

While omitting the UMAP step led to higher classification accuracy, a major advantage of UMAP is the visualization in low-dimensional space, irrespective of the initial number of peptides. To our knowledge, this work is the first of its kind to reduce complex peptidome or proteome data to such a degree that patients of multiple different CKD aetiologies are efficiently presented as single data points in space, forming distinct diagnosis clusters. Dimensionality reduction and visualization properties applied to the urinary peptide information could potentially be used in the context of determining personalized intervention, e.g., drug response prediction. Such an approach may include additional relevant parameters e.g., clinical characteristics, progression, diet, exercise, etc. This hypothesis is currently being investigated in detail and ultimately has to be proven in an appropriately-powered randomized clinical trial.

In the presented approach using a single model for distinguishing multiple CKD aetiologies (instead of multiple models for distinguishing single CKD aetiologies), the overall model performance in the binary classification (DKD, HC) was superior to the multiclass one. This was expected since binary separation is less complex, distinguishing CKD of one aetiology (DKD) from HC. Further, the HC class was distinguished with the highest accuracy in both the binary and multiclass classifications. This can be attributed to the fact that HC participants are pathologically distant from the CKD patients, thus justifiably being distinguishable from the rest of the classes. This observation can be interpreted as further evidence for the validity of the presented approach. In the UMAP-SVM multiclass classification, during predictions on the independent test set, the selected model had the lowest performance distinguishing DKD, assigning a substantial part of its participants to the former majority class, IgAN. Nevertheless, although DKD and IgAN are not that clinically similar, this might be the result of their routine treatment involving several common aspects, especially the anti-hypertension treatment involving angiotensin-converting enzyme inhibitors and angiotensin II receptor blockers, as well as the recently implemented, sodium-glucose cotransporter 2 inhibitors.

Within a biological setting, a plethora of features (e.g., thousands of peptides) detected in a relatively low number of observations (e.g., tens or hundreds, at best) could be an obstacle for a model to identify the relevant underlying pathophysiology patterns for classification (e.g., diagnosis), especially given the common molecular elements between the CKD aetiologies. This situation is well-known in the field as ‘curse of dimensionality’ [34] and in this context, dimensionality reduction algorithms can reduce the number of input features, thus reducing, at least to a degree, the complexity (and potentially irrelevant information that would otherwise ‘confuse’ the model), as well as improving the model's performance. As such, the UMAP [29, 30] algorithm was utilized. However, the SVM model performance in the independent test set was superior when UMAP was not used (∼85% vs ∼70% accuracy, respectively). The reason for this may, among others, be linked to the noise reduction already achieved via applying the 30% sequenced peptide frequency threshold. Using this threshold, out of tens of thousands of peptides detected in urine, 1183 (binary classification) or 1206 (multiclass classification) were considered for further analysis. Using UMAP to further reduce the feature space (and thus the corresponding information contained in the dataset) to only three spatial coordinate features could result in loss of information and thus to a SVM model of reduced (but still noteworthy) performance. Consequently, using UMAP to reduce the feature space could be more useful in cases where an efficient feature selection/removal method is not performed/established. That said, the dimensionality reduction along with its spatial, single-sample, visualization properties constitute UMAP a substantial step in such pipelines. Of note, in the binary classification, the model performances w/o UMAP were similar (∼90% vs ∼96% accuracy, respectively).

Considering that proteomic/peptidomic studies are scarce in CKD differential diagnosis, in the presented study we compared the DKD, IgAN and vasculitis models of the aforementioned earlier study [28], using the CKD273 scoring to define the HC group [25]. As expected from the anticipated difference in molecular pathology, the HC class could be separated with the highest accuracy. In comparison with these single-aetiology models, comparable or slightly improved performance was observed using our presented approach. Nevertheless, the apparent superior performance in terms of the vasculitis class should be viewed with caution since only six vasculitis patients were tested in this earlier single-aetiology model.

The presented study has limitations. First, class balance was not the case for the multiclass classification data. Class imbalance is hardly avoidable when working with retrospective datasets, among other things due to the inherent difference in disease prevalence. We attempted to address this issue by introducing randomly synthetic participants to each class using an algorithm [31], but larger studies, ideally of equally-numbered classes are warranted. Furthermore, the CKD aetiologies investigated herein represent only a fraction (nevertheless, the majority) of the broad CKD spectrum, and thus the inclusion of additional aetiologies in further studies seems well justified. It is also expected that the inclusion of relevant clinical parameters may increase model performance. However, due to incomplete clinical records of some participants, this could not be implemented in the presented study. Additionally, since UMAP is not as interpretable as e.g., principal component analysis, cluster sizes in UMAP plots and distances between the clusters as well as the potential impact of random noise, among others, entail caveats that could potentially result in misinterpreting the plots [33]. Lastly, only the SVM (radial basis kernel) classifier was assessed; the performance of other machine learning algorithms may even be superior.

Kidney biopsy can be utilized to acquire information not only on CKD aetiology, but also in terms of disease severity, subclassification, chronicity and co-existing conditions. Nevertheless, artificial intelligence tools developed through the presented pipeline could theoretically be trained to deliver such information, with the advantage, due to the non-invasive approach, of being applied multiple times, consequently enabling monitoring of disease progression and guiding towards optimal therapeutic decisions.

In conclusion, in this proof-of-concept study, we established a robust pipeline for simultaneous classification of multiple CKD aetiologies and sample visualization in the 3D space based on urinary peptides. The approach enables discrimination of major different CKD aetiologies and can be used to establish differential diagnosis without the need to perform an invasive kidney biopsy, which may be especially relevant in early detection. We anticipate that this will also serve as a basis for developing models as supplementary clinical tools, enabling the assessment of additional CKD aetiologies and also other diseases.

## Supplementary Material

gfad200_Supplemental_File

## Data Availability

Data will be made available upon request directed to the corresponding author. Proposals will be reviewed and approved by the investigators and collaborators based on scientific merit. After approval of a proposal, data will be shared through a secure online platform after signing the data access and confidentiality agreement. Code was generated based on the functions in the respective R packages as described in the methods and will be made available upon request directed to the corresponding author.
